# Toward an Understanding of the Pressure Effect on the Intramolecular Vibrational Frequencies of Sulfur Hexafluoride

**DOI:** 10.1021/acs.jpca.1c02595

**Published:** 2021-07-15

**Authors:** Matteo Boccalini, Roberto Cammi, Marco Pagliai, Gianni Cardini, Vincenzo Schettino

**Affiliations:** † Dipartimento di Chimica “Ugo Schiff”, Università degli Studi di Firenze, Via della Lastruccia 3, 50019 Sesto Fiorentino, Italy; ‡ Dipartimento di Scienze Chimiche, della Vita e della Sostenibilità Ambientale, Universitá degli Studi di Parma, Parco Area delle Scienze 11/a, 43124 Parma, Italy

## Abstract

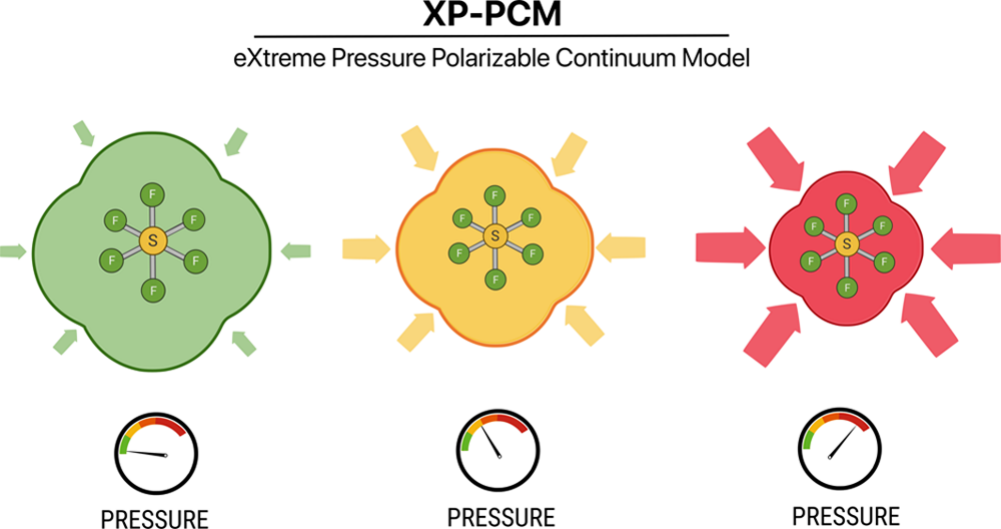

The structural and vibrational properties of the molecular units of sulfur hexafluoride crystal as a function of pressure have been studied by the Extreme Pressure Polarizable Continuum Model (XP-PCM) method. Within the XP-PCM model, single molecule calculations allow a consistent interpretation of the experimental measurements when considering the effect of pressure on both the molecular structure and the vibrational normal modes. This peculiar aspect of XP-PCM provides a detailed description of the electronic origin of normal modes variations with pressure, via the curvature of the potential energy surface and via the anharmonicity of the normal modes. When applied to the vibrational properties of the sulfur hexafluoride crystal, the XP-PCM method reveals a hitherto unknown interpretation of the effects of the pressure on the vibrational normal modes of the molecular units of this crystal.

## Introduction

The study of structural and dynamic properties of both liquid and solid materials at high pressure, mainly carried out in the diamond anvil cell (DAC),^1−3^ is an active research field^1,2,4−7^ for its implication in both applied science and technology and knowledge of atomic and molecular properties. Indeed, in a recent paper by Cammi, Rahm, Hoffmann and Ashcroft^8^ it has been envisaged that basic electronic properties along the periodic table (like atomic electronic configuration and electronegativity) exhibit drastic changes at high pressures thus modulating general chemical behaviors.

In particular, in studying molecular crystals at high pressures, the general properties can be conveniently investigated with vibrational spectroscopy and some experimental findings can be achieved by comparison with computational models. A general approach to model molecular crystals involves periodic systems.^9−12^ Accurate determination of structural and spectroscopic properties by *ab initio* solid state calculations requires elevated computational resources.^10,11^ But, even when vibrational properties are obtained either by *ab initio* molecular dynamics simulations or by crystal calculations in the harmonic approximation^10−12^ subsequent extraction of high pressure effects on single molecule is not an easy task.

The recently introduced *eXtreme Pressure Polarizable Continuum Model* (XP-PCM)^4,13−18^ allows for *ab initio* calculations at high pressure on a single molecular unit of the system of interest at a reduced computational cost. In the XP-PCM calculations the main pressure effects on the vibrational properties can be easily taken into account and these are interpreted in terms of curvature and anharmonicity of the potential energy surface, providing useful insights on pressure effects on the intramolecular normal modes,^16−18^ a kind of analysis which is not currently available in periodic calculations. In the XP-PCM approach the interactions with the environment are modeled using the Polarizable Continuum Model (PCM),^19,20^ properly taking into account the pressure effect by increasing the Pauli repulsion due to the external medium on the molecule.^4^ This tuning of the pressure is obtained by reducing the volume of the cavity hosting the molecular unit.^4,13,16,17,21^

**Figure 1:**
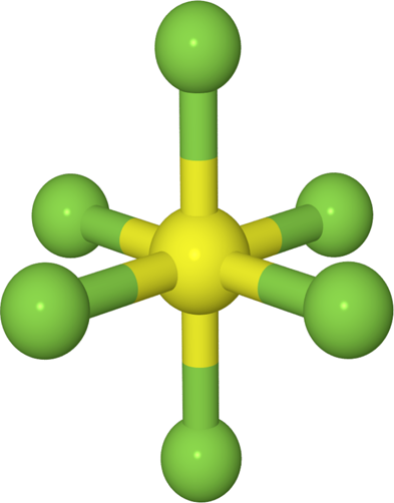
Molecular structure of sulfur hexafluoride SF_6_.

In the present paper the XP-PCM method has been applied to study the vibrational frequencies of sulfur hexafluoride (SF_6_) as a function of the pressure and how these effects can be properly analyzed. Sulfur hexafluoride is an interesting candidate, since the molecular structure of isolated SF_6_, as depicted in Figure 1, has a perfect octahedral arrangement (almost spherical). The high symmetry of the SF_6_ molecule (*O*_*h*_) and the limited number of atoms allow to analyze the effect of pressure on vibrational properties at high level of theory and to compare the results with the available experimental measurements.^22,23^ In fact, several IR and Raman experiments^22−26^ are available on the molecular crystal for different phases both at ambient and high pressures.^23,26^

The article is organized as follows. First, the XP-PCM theory is briefly reviewed in the “XP-PCM Theory” section, then the phase diagram is discussed in the “Phase Diagram of Sulfur Hexafluoride” section. The computational procedure is described in the “Computational Protocol and Details” section and finally the results of the calculations are presented in the “Results and Discussion” section, where a detailed analysis of the vibrational properties in terms of curvature and relaxation contributions are discussed. Final remarks are reported in the “Conclusions” section.

## Methods

### XP-PCM Theory

Let us consider a molecular systems embedded in the external medium of the XP-PCM model. The electronic energy determining its potential energy surface is given by eq 1
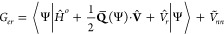
1where *Ṽ*_*nn*_ is the nuclei–nuclei interaction contribution in the presence of the external medium, **Q̅**(Ψ)·**V̂** is the PCM electrostatic interaction term^20^ and *V̂*_*r*_ is the Pauli repulsion term, which describes the exchange–repulsion contribution due to the overlap of the electron distribution of the studied molecular system with the mean electron distribution of the external medium (modeled by an uniform distribution outside the cavity following the PCM approach^19,20^). The repulsion term is expressed by eq 2
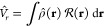
2where ρ̂(**r**) = ∑_*i*_^*n*^δ(**r** – **r**_*i*_) is the electron density operator (over the *n* electrons of the molecular system).

 is a factor representing a step barrier potential located at the boundary of the molecular cavity, as reported in eq 3:
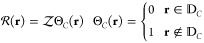
3where  is the height of the potential barrier and Θ_*C*_(**r**) is a generalized Heaviside step function with a value equal to zero inside the cavity and equal to one outside of it ( denotes the domain of the physical space inside the cavity). The height of the potential barrier  depends on the volume of the cavity, *V*_*c*_,^4^ as shown in eq 4:
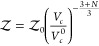
4where  is the height corresponding to a reference upper value of the cavity volume, *V*_*c*_^0^, and *N* is a semiempirical parameter that gauges the strength of the Pauli repulsive barrier originated from the external medium, and which can be estimated by comparison of the computed pressure–volume results of XP-PCM with the available experimental pressure–volume data as expressed by Murnaghan equation^27^ for different phases. The height of the potential barrier  given in eq 4 implicitly accounts for the pressure dependence of the properties of the external medium^13^ (i.e., the dielectric permittivity and average electron density).

The pressure *p* is computed as the negative of the derivative of the electronic energy, eq 5, with respect to the cavity volume *V*_*c*_ at constant number of particles *n*:
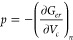
5

The cavity is defined in terms of overlapping spheres centered on the atomic nuclei with radius related, by a uniform scaling factor (*f*), to the corresponding atomic van der Waals radii (*R*_*vdW*_^*i*^).^28^ The cavity volume may be freely reduced by decreasing the scaling factor with respect to the reference value *f*^0^ = 1.2.^4,13,16−18^ It has been recently shown^29^ that the XP-PCM model is able to faithfully reduce the van der Waals radii of elements in agreement with available experimental data, suggesting that the same procedure can be successfully extended in studying molecular species.

The Pauli repulsion potential depends indirectly on *f*. In fact, the repulsion potential is related both to the numeral density of the external medium  and by a semiempirical parameter , with 3 ≤ *N* ≤ 6, which can be experimentally determined, where *a*_0_ and *E*_*h*_ are conversion factors from Bohr and Hartree, respectively.

The equilibrium geometries of the molecule under pressure correspond to the minimum of the potential energy surface determined by the electronic functional *G*_*er*_ of eq 1.

The vibrational harmonic frequencies are obtained from the Hessian matrix of the second derivatives of *G*_*er*_ with respect to the atomic displacements obtained numerically by the gradients.

As mentioned in the Introduction, the XP-PCM helps to achieve relevant insights on the pressure effect on vibrational frequencies by a partition in curvature and relaxation (or anharmonicity) contributions;^4,16,17^ these contributions are referred to also as direct and indirect effects, respectively.

### The Effect of the Pressure on the Equilibrium Geometry and Vibrational Frequencies and Its Direct and Indirect Contributions

Describing the molecule as a set of harmonic oscillators, the electronic energy *G*_*er*_ in eq 5 can be expanded as a function of the vibrational normal mode coordinates of the isolated molecule **Q** and of the pressure *p*, as shown in the Taylor expansion in eq 6:
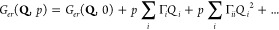
6where *G*_*er*_(**Q**, 0) is the electronic energy at zero pressure (i.e., that of the isolated molecule), as a function of the normal coordinates **Q**; Γ_*i*_ and Γ_*ii*_ are partial derivatives of *G*_*er*_ and their physical meaning is summarized in the following.

Equation 7 shows that Γ_*i*_ is the mixed second order derivative:
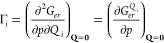
7and it represents the direct effect of pressure on the forces on the nuclei along the normal coordinate Q_*i*_. Due to the isotropic pressure, Γ_*i*_ is forced to be different from zero only for totally symmetric (TS) normal mode coordinates. In particular, the variations of the equilibrium geometry along the TS normal coordinates {*Q*_*i*_^*TS*^} are given by eq 8
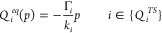
8where *k*_*i*_ is the harmonic force constants of the *i*^*th*^ normal mode. Equation 8 shows also how the pressure affects the geometry not only through the coupling coefficients {Γ_*i*_} but also with the normal mode force constants, {*k*_*i*_}, which describe the stiffness of the normal modes.

The Γ_*ii*_ is expressed in eq 9 by the mixed third-order coefficient:
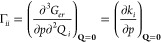
9and it represents the direct effect of the pressure on the force constant *k*_*i*_ of the *i*^*th*^ normal mode. Γ_*ii*_ could be different from zero for any vibrational normal mode.

The total effect of pressure on the vibrational harmonic force constants is given by eq 10:
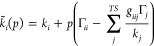
10where *g*_*iij*_ is the cubic anharmonic constant coupling the normal mode *i* with a totally symmetric normal mode *j*.

Furthermore, the effect of the pressure on the vibrational frequencies can be partitioned into a curvature (direct) and a relaxation (indirect) contribution,^4,13,16,17^ as shown in eq 11:
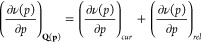
11with
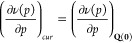
12
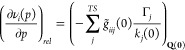
13where ν(*p*) denotes the vibrational frequencies evaluated at the equilibrium geometries and **Q**(0) the equilibrium geometry of the isolated molecule.

From eqs 11–13, the pressure effect on the harmonic force constants can be partitioned in a *curvature (or direct)* effect, represented by Γ_*ii*_, which relates the influence of pressure to changes in the electron density of the system, and a *relaxation (or indirect)* effect, involving the cubic anharmonicities *g*_*iij*_, which is related to the pressure induced modification of the equilibrium geometry of the molecular system.

Equations 11–13 are of fundamental importance to analyze the effect of pressure on vibrational properties of molecules in the crystal. In essence, the *curvature* contribution, due to the variation of the second derivative of the potential energy surface, accounts for the confinement effect due to the repulsive intermolecular interactions, evaluated at the zero-pressure equilibrium geometry, while the *relaxation* contribution is related to the variation of the equilibrium geometry of the molecule through the anharmonic force constants. In more general terms, this analysis is motivated by the need to give answer to some questions like “Why do the molecules respond to the pressure in the way observed? Why does this response depend on the various vibrational normal modes? Is there a property of the normal modes that governs their response to the pressure?”. We remark that a similar partitioning, useful to rationalize observed frequency shifts with pressure, has previously been carried out with a semiclassical approach by Moroni et al.^30^ The XP-PCM method has the definite advantage of accurately estimating the two contributions for a general molecular system.

## Phase Diagram of Sulfur Hexafluoride

The phase diagram of SF_6_ has been studied as a function of both temperature and pressure. At ambient pressure it has been found by neutron diffraction experiments^31^ that at 94.3 K a phase transition occurs from a high temperature orientationally disordered structure (space group *Im*3*m*, *Z* = 2) to a monoclinic structure (space group *C*_2_/*m* – *C*_2*h*_^3^ with *Z* = 6). The molecules are found in two different sites, with site symmetry 2/*m* (or *C*_2*h*_) or *m* (or *C*_*s*_). The reduction of molecular symmetry and the intermolecular interactions in the crystal produce a multiplet structure in the vibrational spectra. The correlation diagram for the *C*_2*h*_ site shows that the degeneracy of the normal modes is removed. Therefore, for molecules on this site it is expected that the *A*_1*g*_, *E*_*g*_, and *T*_2*g*_ should appear as singlet, doublet, and triplet, respectively, in the Raman spectrum. The same turns out to be the case for the two molecules on the *C*_*s*_ site since an additional Davydov component is only infrared active. For this latter lattice site each component of the infrared modes should become Raman active. In the Raman spectrum of Salvi and Schettino^22^ the *A*_1*g*_ mode shows two components which should be taken as arising from the *C*_2*h*_ and *C*_*s*_ sites. For the *E*_*g*_ and *T*_2*g*_ modes 4 and 3 components are observed, respectively, and could, by analogy, be taken as arising from the molecules on different sites. No evidence is found of Raman activation of the infrared modes or of infrared activation of the Raman modes. This implies that in the low temperature phase the molecular deformation of the SF_6_ octahedra is actually very small. The model used in the present paper appears, therefore, suited for the system under study. As a whole the internal Raman active components should classify as 14 *A*_*g*_ ⊕ 7 *B*_*g*_ in the factor group.

The SF_6_ phase diagram as a function of pressure has been explored by X-ray diffraction up to 32 GPa.^23^ A phase transition to the same crystal structure of the low temperature phase described by Cockcroft and Fitch^31^ has been observed at 2 GPa (phase II). Further transitions have been observed to occur at 12 GPa (phase III) and 24 GPa (phase IV) as evidenced by the appearance of new lines in the diffraction pattern and changes in the Raman spectrum. It can be remarked that the multiplet structure observed in phase III is identical with that reported by Salvi and Schettino at low temperature and ambient pressure,^22^ apart from the improved resolution of the low temperature spectrum. It can be argued that also at high pressure deviations from the octahedral structure of the SF_6_ units are very small. Therefore, a geometry belonging to the *O*_*h*_ point group symmetry^32−35^ has been adopted in the initial configuration of DFT calculations. Subsequently, the pressure effects on both the structural and vibrational properties of SF_6_ have been simulated by increasing the Pauli exchange repulsion experienced by the target molecule properly reducing the cavity volume using the XP-PCM method.^4,13,16−18^

### Computational Protocol and Details

All the calculations have been performed in the DFT framework adopting the 6-311G(d)^36^ basis set with a modified version of the Gaussian 09 rev.C.01 suite of programs:^37^ The exchange–correlation energy functional has been selected by verifying the accuracy in reproducing the experimental S–F bond length with some exchange and correlation functionals.

The bond length of the optimized SF_6_ molecule (in gas phase) obtained with the selected exchange and correlation functional is reported in Table 1; the computed bond lengths are in the range between 1.588 Å (PBE0 and WB97XD) and 1.638 Å (BLYP).

**Table 1:** Bond Length (Å) of Isolated SF_6_ Molecule for Selected Exchange and Correlation Functionals, Using the 6-311Gd) Basis Set^a^

functional	S–F (Å)
exp^38^	1.561
SVWN^39−42^	1.591
BLYP^43,44^	1.638
B3LYP^44,45^	1.603
CAM-B3LYP^46^	1.589
O3LYP^47^	1.600
PBE^48,49^	1.622
PBE0^50^	1.588
WB97XD^51^	1.588

a The computed SF_6_ bond length is compared with experimental data in the gas phase.^38^

PBE0 and WB97XD provide results in better agreement with experiments.^38^ Therefore, we have decided to report the structural and spectroscopic analysis on the basis of calculations with the PBE0 functional, unless otherwise specified.

The XP-PCM protocol is the same employed with success in the studies on P_4_S_3_^17^ and on As_4_S_4_.^18^ Argon has been used as solvent, while van der Waals radius for S and F have been set to 1.80 and 1.47 Å, respectively.^28^ The scaling factor *f* has been varied between 1.2 and 0.975, allowing to study the system in the pressure range between 0 and 10.2 GPa for *N* = 3 and between 0.0 and 24.6 GPa for *N* = 6, respectively. To ensure a smooth behavior of electronic and structural properties with pressure, the molecular cavities have been described using tesserae with an average size of 0.05 Å^2^ (the value has been imposed with the keyword TSARE and it is about an order of magnitude lower than the default value which is 0.2 Å^2^). However, it has been verified that a value of 0.075 Å^2^ represents a good compromise between accuracy and computational time for the present calculations.

Analogously to XP-PCM studies on P_4_S_3_,^17^ and As_4_S_4_,^18^ the protocol consists in a molecular geometry optimization performed with a self-consistent procedure until the structure convergence is reached for each scaling factor *f* (i.e., pressure value). Once the equilibrium geometry has been located, the procedure is repeated for a new cavity with a smaller scaling factor. The convergence criteria imposed in the calculations are *Maximum force* lower than 2 × 10^–6^*E*_*h*_/*a*_0_, *RMS force* lower than 1 × 10^–6^*E*_*h*_/*a*_0_, *Maximum atomic displacement* lower than 6 × 10^–6^*a*_0_ and *RMS atomic displacement* lower than 4 × 10^–6^*a*_0_. An *ultrafine* grid has been adopted for the evaluation of the numerical integrals. All the calculations on SF_6_ have been performed with values for the *N* parameter equal to both 3 and 6, as discussed in the following. This choice of using the limiting values for *N* is based on previous works^4,13,15−17,52^ on XP-PCM that proved how it was possible to interpret the experimental observable with pressure. No fitting procedures have been carried out to further improve the agreement between experimental and computed data.

The harmonic vibrational frequency calculation has been carried out for all the optimized geometries. The pressure effects on vibrational normal modes have been analyzed to determine both the *curvature* and *relaxation* contributions. The *curvature* contribution, eq 12, has been evaluated through the calculation of the vibrational frequencies, obtained by numerical differentiation, as a function of pressure at fixed equilibrium geometry *in vacuo*. The *relaxation* contribution can be evaluated numerically by eq 11 or analytically by determining the derivative of frequency variation with pressure as in eq 13.

## Results and Discussion

### IR and Raman Spectra at Ambient Pressure

The isolated SF_6_ molecule belongs to the *O*_*h*_ point group and consequently the vibrational normal modes can be grouped in the irreducible representations as
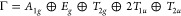
14

The normal modes with symmetry *A*_1*g*_, *E*_*g*_, and *T*_2*g*_ are Raman active, whereas the two *T*_1*u*_ modes are IR active. The normal mode of *T*_2*u*_ symmetry is both IR and Raman inactive, but it has been identified and assigned by Salvi et al.^22^ analyzing combination bands in the IR spectrum of the crystal.

Calculated and experimental frequencies are compared in Table 2. It can be noted that the scaled calculated frequencies agree quite satisfactorily with the crystal frequencies.^22,23^ This may imply that the XP-PCM approach somehow accounts for the static field acting on the molecule albeit not (obviously) for the intermolecular coupling giving rise to the Davydov splitting.

**Table 2:** Normal Modes (cm^–1^) of SF_6_^a^

symm	NM	scaled	exp^b^	*Δν*	%	free	assignment
*T*_2*u*_	328.2	346.9	(345.7)^c^	1.2	0.3	326.9	ν_6_, antisymm degenerate bending
*T*_2*g*_	489.6	517.5	523.5	6	1.2	488.7	ν_5_, symm degenerate bending
*T*_1*u*_	578.3	611.3	(611)	0.3	0.05	578.9	ν_4_, antisymm degenerate bending
*E*_*g*_	626.7	662.4	644	18.4	2.9	627.1	ν_2_, symm degenerate stretching
*A*_1*g*_	729.6	771.2	775	3.8	0.5	728.0	ν_1_, symm stretching
*T*_1*u*_	925.7	978.4	(936)	42.4	4.5	934.2	ν_3_, antisymm degenerate stretching

a XP-PCM results with *f* = 1.20 at the PBE0/6-311G(d) level of theory. TSARE = 0.05. Vibrational frequencies (NM) have been scaled by a factor of 1.057, obtained through a fitting procedure for the calculated frequencies of the isolated molecule (without XP-PCM).

b P.R. Salvi and V. Schettino.^22^

c This frequency has been obtained from the analysis of combination bands. *Δν* represents the absolute error in cm^–1^, while with % are reported the absolute error in percentage. The assignment has been taken from Herzberg.^53^ Symmetries for the “free” molecule calculated frequencies at the same XP-PCM level of theory are reported.

The vibrational assignment is that reported by Herzberg^53^ and confirmed in several studies of the vibrational properties of SF_6_.^22−26,54,55^ In particular, the three lower vibrational frequencies are bending normal modes, whereas the others are stretching modes.

**Figure 2:**
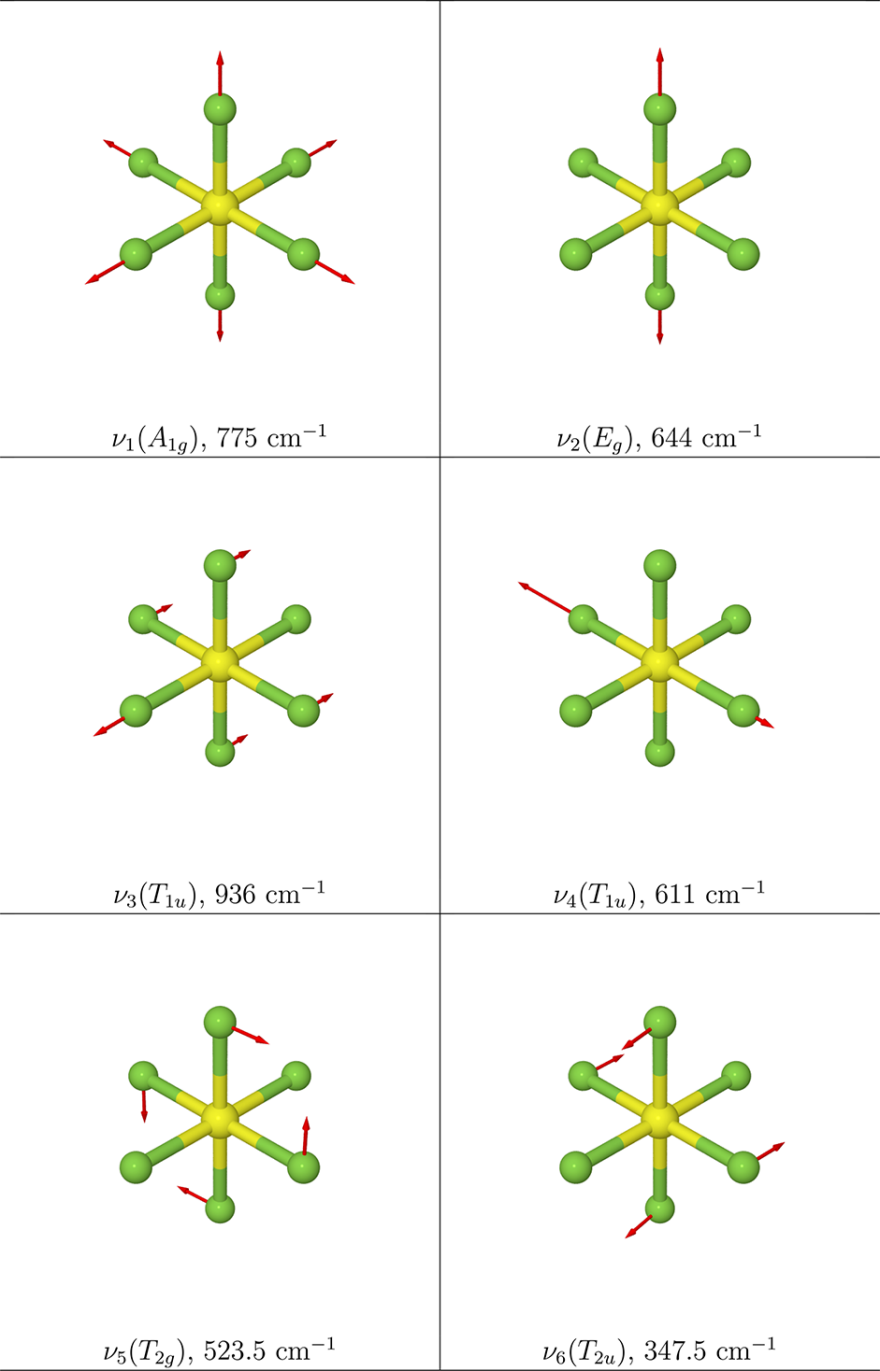
Graphical representation of the normal modes of SF_6_ with the assignment proposed by Salvi et al.^22^ Only one component of the each degenerate modes is shown.

A graphical representation of the normal modes is reported in Figure 2.

### Structural Properties

The first information on the response to pressure of SF_6_ molecule has been achieved by computing the Murnaghan^27^ and Birch–Murnaghan^56^ equation of state and comparing the results with experimental data.^23^

The relation between the scaling factor *f* and the pressure can be obtained assuming that the cavity volume can be initially described through the Murnaghan equation:^27^
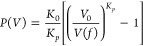
15in which *V*_0_ is the cavity volume for *f* = 1.20 and *V*(*f*) is the cavity volume for a certain scaling factor, whereas  and .

The *K*_0_ and *K*_*p*_ parameters are obtained by using a fitting procedure of the electronic energy functional:
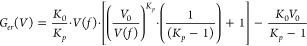
16

A similar procedure has been performed using the Birch–Murnaghan equation of state:^56^
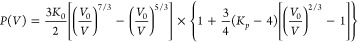
17

The values of both the *K*_0_ and *K*_*p*_ Murnaghan parameters obtained by XP-PCM calculations are compared in Table 3 with experimental data by Rademacher et al.^23^ for both phase I and phase II. The XP-PCM calculations have been carried out with the *N* parameter equal to both 3 and 6. The Birch–Murnaghan equation of state obtained for phase II by Rademacher et al.^23^ with periodic DFT calculations (PBE+TS) provides results in excellent agreement with experiments.

**Table 3:** Comparison of *K*_0_ and *K*_*p*_ Murnaghan Parameters Obtained by XP-PCM Calculations on SF_6_ and Experiments^23^

	*N* = 3	*N* = 6	exp phase I	exp phase II
*K*_0_	6.46	7.79	6.3(2)	8.5(8)
*K*_*p*_	5.01	7.19	4	7.4(9)

**Figure 3:**
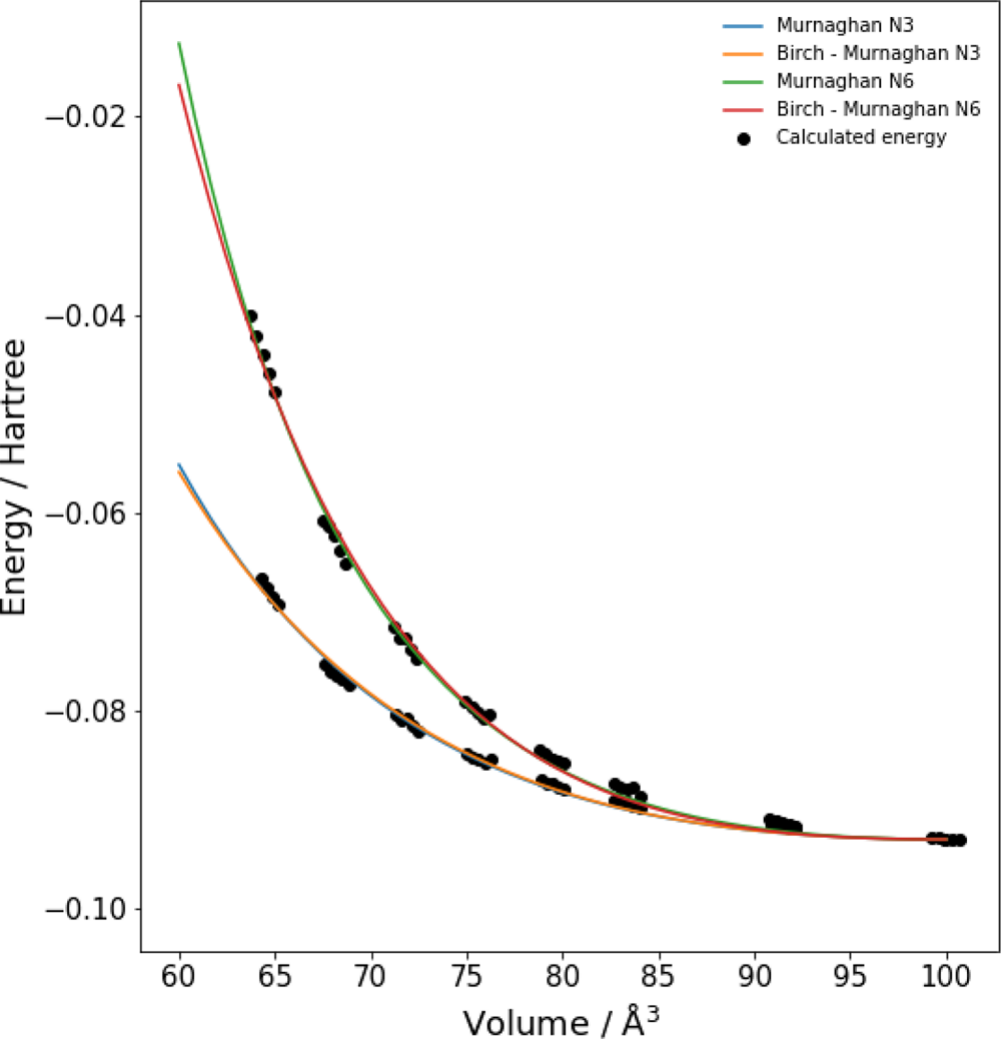
Fit of energy values with both Murnaghan^27^ and Birch–Murnaghan^56^ equations of state. The procedure has been carried out for XP-PCM calculations with both *N* = 3 and *N* = 6.

The comparison of the fitting procedure with both the Murnaghan^27^ and Birch–Murnaghan^56^ equations of state is reported in Figure 3. It is possible to appreciate from the Figure 3 that the curves of the energy as function of the cavity volume are accurately reproduced with both the Murnaghan^27^ and Birch–Murnaghan^56^ equations of state, with appreciable differences only outside the range of the computed data (cavity volume <65 Å^3^).

The fitting procedure has been carried out employing both the *N* = 3 and *N* = 6 values in the XP-PCM calculations, as in Table 3.

**Figure 4:**
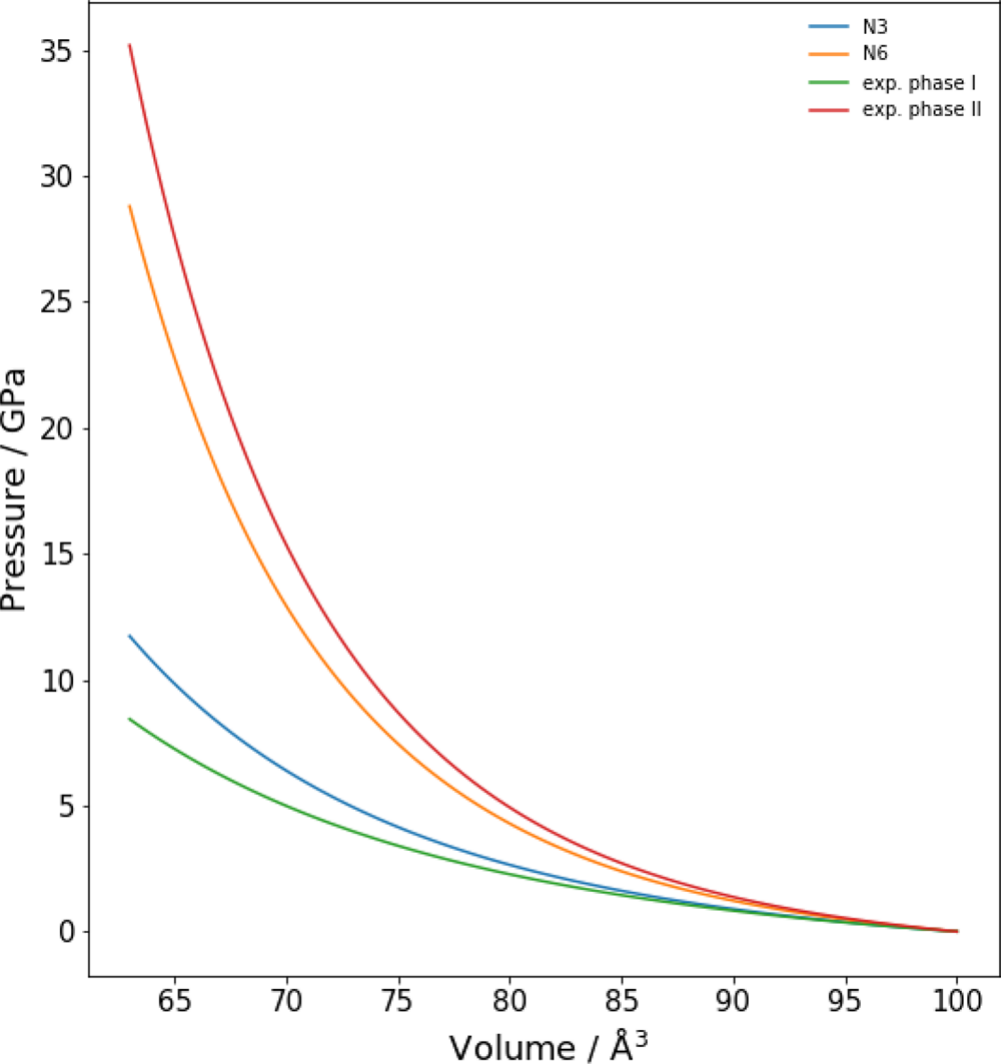
Comparison between experimental and computed Murnaghan^27^ equation of states with volume. Cavity volume and molecular volume have been adopted for calculations and experiments, respectively.

Since the SF_6_ molecular crystal undergoes phase transitions with pressure,^23^ an appropriate tuning of the *N* parameter allows to follow correctly the structural behavior in different pressure ranges, as it can be appreciated in Figure 4 where both computed and experimental Murnaghan^27^ equations of state are reported. The computed Murnaghan equation of state with *N* = 3 closely resemble the experimental one for phase I, whereas the XP-PCM calculations with *N* = 6 reproduce the behavior of phase II.

### Pressure Effects on Bond Lengths

**Figure 5:**
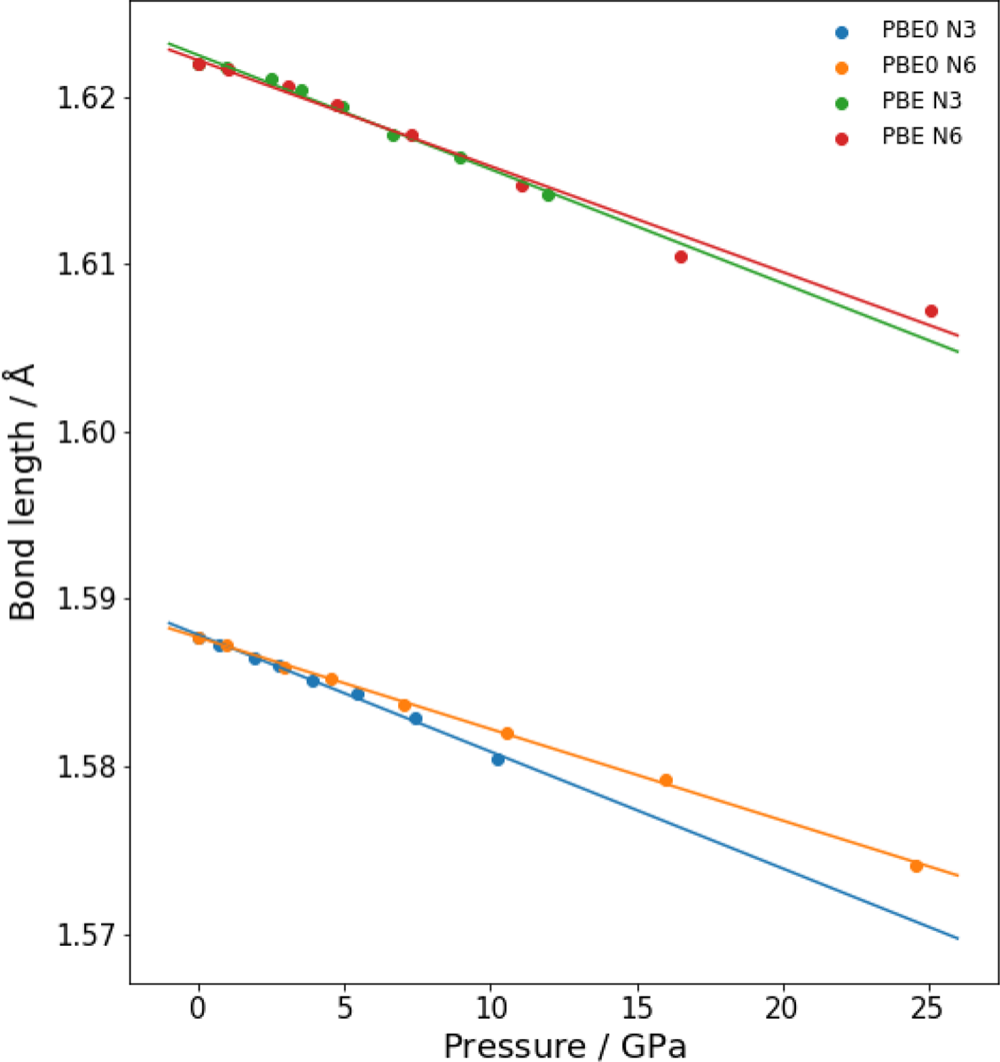
S–F bond length (in Å) with the pressure (in GPa). The results are reported for both PBE and PBE0 exchange and correlation functionals in conjunction with 6-311G(d) basis set. The equations of linear regression are PBE N3, *y* = −0.00068*x* + 1.62246; PBE N6, *y* = −0.00063*x* + 1.62216; PBE0 N3, *y* = −0.00070*x* + 1.58786; and PBE0 N6, *y* = −0.00055*x* + 1.58770.

The different effect due to the *N* parameter with increasing pressure provides further information on the compressibility of SF_6_. It is interesting to note (see Figure 5) that *N* = 3 and *N* = 6 parameters present a similar behavior for low pressure values, whereas a marked difference occurs for higher values, as confirmed by linear regression analysis. In fact, the correlation coefficients for the linear regression equations for PBE are *R*^2^ = 0.9907 and *R*^2^ = 0.9870 for *N* = 3 and *N* = 6, respectively, while the same coefficients for PBE0 are *R*^2^ = 0.9923 and *R*^2^ = 0.9989. By comparing the results obtained with both the PBE0 and PBE exchange and correlation functionals, it is possible to note that hybrid functionals provide a better agreement than GGA functionals with the experimental bond length^38^ and a similar trend with pressure. The bond length contraction has the same order of magnitude, but it presents a slightly underestimated value.^23^ Although marked volume variations are observed with the choice of *N* parameter as shown in Figure 3, the variation of the bond lengths are less affected by the *N* value. This behavior reflects on the calculated frequencies which are usually more accurate with PBE0 than PBE. This result further suggests to use hybrid functionals in simulation of structural and vibrational properties by performing calculations with the XP-PCM method.

As previously observed in studying other systems with the XP-PCM method,^4,13,16,17^ the effect of pressure on SF_6_ molecular structure can be analyzed considering eq 8. In fact, the variation of molecular structure with pressure are related both to pressure coupling coefficient Γ_*i*_ and to the stiffness of the normal modes. This analysis can be carried out through two different approaches. In the first case, the pressure coupling Γ_*i*_ (defined in eq 7) is estimated by a linear fitting of the nuclear gradient of *G*_*er*_ as function of pressure evaluated at the equilibrium geometry of the isolated molecule. The force constant *k*_*i*_ is determined from the calculation of the harmonic frequencies of the isolated molecule. In the second case, the linear coefficient () has been obtained by the projection of the atomic displacement on the eigenvector of the totally symmetric normal mode, as function of pressure. The two procedures have been performed for *N* = 3 and *N* = 6, and the final results, collected in Table 4, allow to verify eq 8.

**Table 4:** Pressure Coupling Constant Γ_*i*_ (*E*_*h*_*a*_0_^–1^ GPa^–1^), Defined in Eq 7, Harmonic Force Constant in the Gas Phase *k*_*i*_ (*E*_*h*_*a*_0_^–2^), Ratio Γ_*i*_/*k*_*i*_ (Å GPa^–1^), and Pressure Coefficient d*Q*_*i*_/d*p* (Å GPa^–1^) of SF_6_

	Γ_*i*_	*k*_*i*_		
*N* = 3	0.00123	0.7201	–0.00173	–0.00171
*N* = 6	0.00099	0.7201	–0.00137	–0.00134

### Pressure Effects on the Vibrational Modes

The pressure effects on the vibrational frequencies have been obtained from calculations for the SF_6_ pressure optimized geometrical structures (scaling factor, *f*) for both the *N* = 3 and *N* = 6 parameters. The vibrational frequencies of the computed normal modes of SF_6_ as a function of pressure are collected in Table 5 and 6.

**Table 5:** Calculated Frequencies (in cm^–1^) at the Different Pressure Values (GPa) with Parameter *N* = 3

symmetry	0.0	0.7	1.9	2.8	3.9	5.4	7.4	10.2
*T*_2*u*_	346.9	348.4	350.9	352.9	355.7	359.2	364.2	371.2
*T*_2*g*_	517.5	518.6	520.8	522.3	524.6	527.3	531.5	537.7
*T*_1*u*_	611.3	611.7	612.8	613.6	615.0	616.5	619.1	622.8
*E*_*g*_	662.4	663.3	665.5	666.5	668.8	671.4	675.7	682.1
*A*_1*g*_	771.2	772.6	775.5	777.1	780.0	783.0	788.0	795.7
*T*_1*u*_	978.5	977.3	977.2	976.7	977.5	978.0	980.2	985.1

**Table 6:** Calculated Frequencies at the Different Pressure Values (GPa) with Parameter *N* = 6

symmetry	0.0	1.0	3.0	4.6	7.0	10.6	16.0	24.6
*T*_2*u*_	346.9	348.7	352.4	355.6	360.6	367.2	377.0	391.6
*T*_2*g*_	517.5	518.8	521.9	524.5	528.7	534.0	542.3	555.3
*T*_1*u*_	611.3	611.9	613.8	615.3	618.0	621.5	627.1	635.6
*E*_*g*_	662.4	663.6	667.0	669.3	673.7	679.3	688.5	702.8
*A*_1*g*_	771.2	772.9	777.2	779.7	785.0	791.2	801.1	817.3
*T*_1*u*_	978.5	977.6	978.6	979.1	982.2	985.4	992.3	1005.8

#### Comparison with Experiments

**Figure 6:**
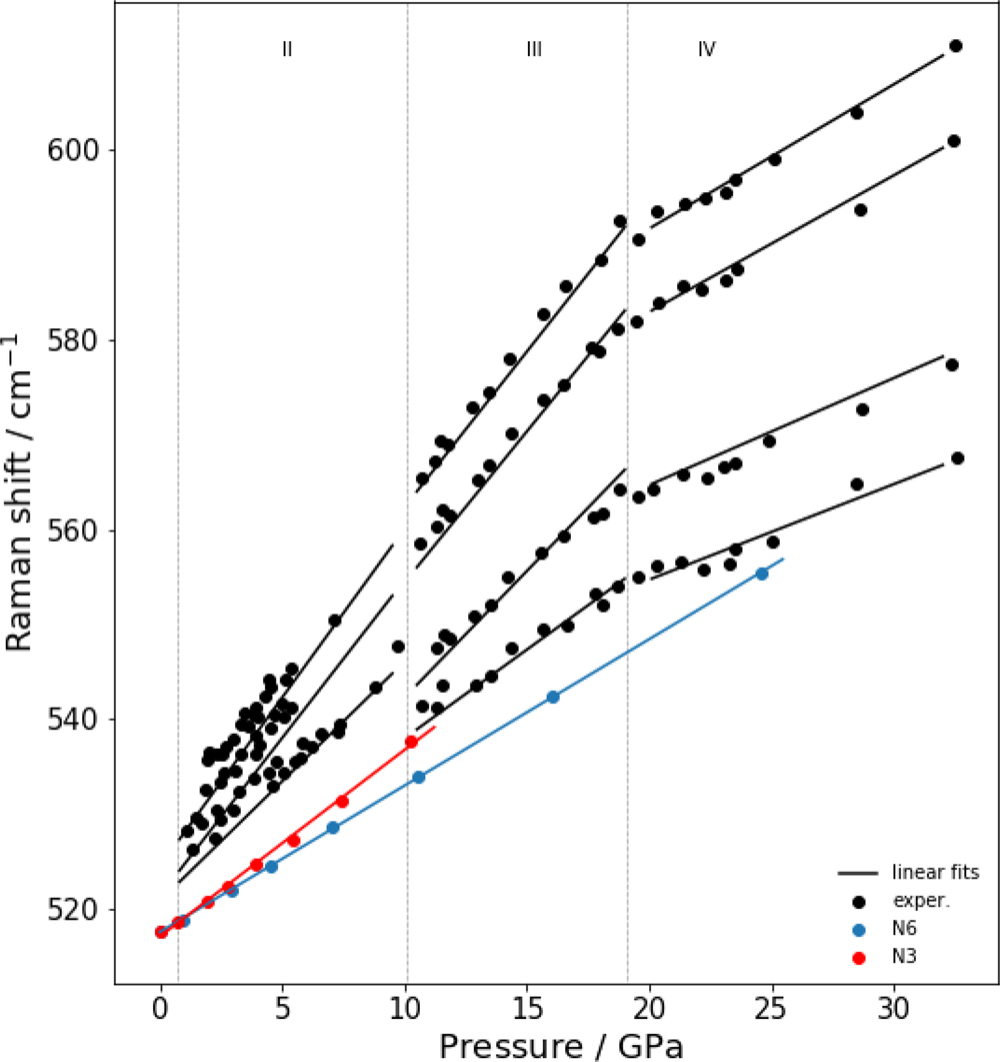
Experimental and computed vibrational frequencies of normal mode ν_5_(*T*_2*g*_). The XP-PCM calculations have been carried out with both *N* = 3 and *N* = 6. The experimental data have been taken by Rademacher et al.^23^

**Figure 7:**
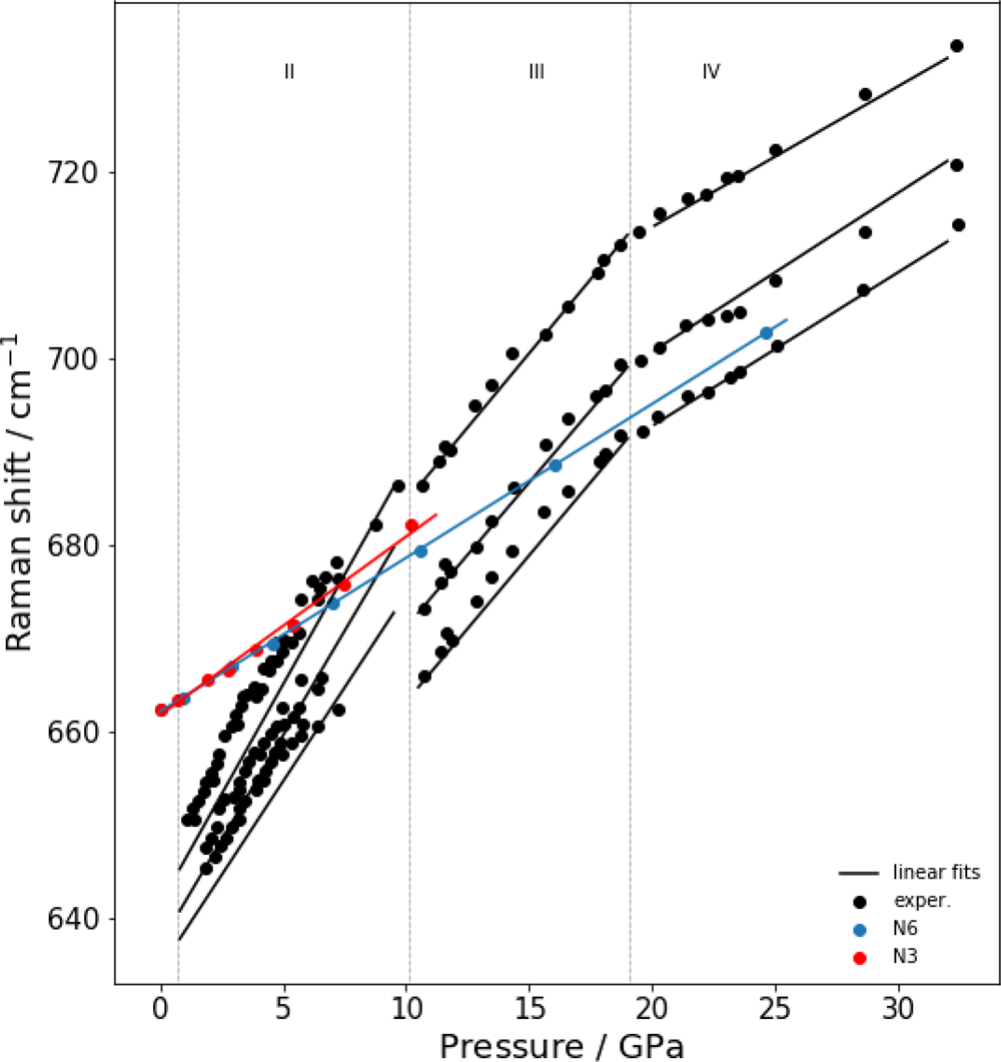
Experimental and computed vibrational frequencies of normal mode ν_2_(*E*_*g*_). The XP-PCM calculations have been carried out with both *N* = 3 and *N* = 6. The experimental data have been taken by Rademacher et al.^23^

**Figure 8:**
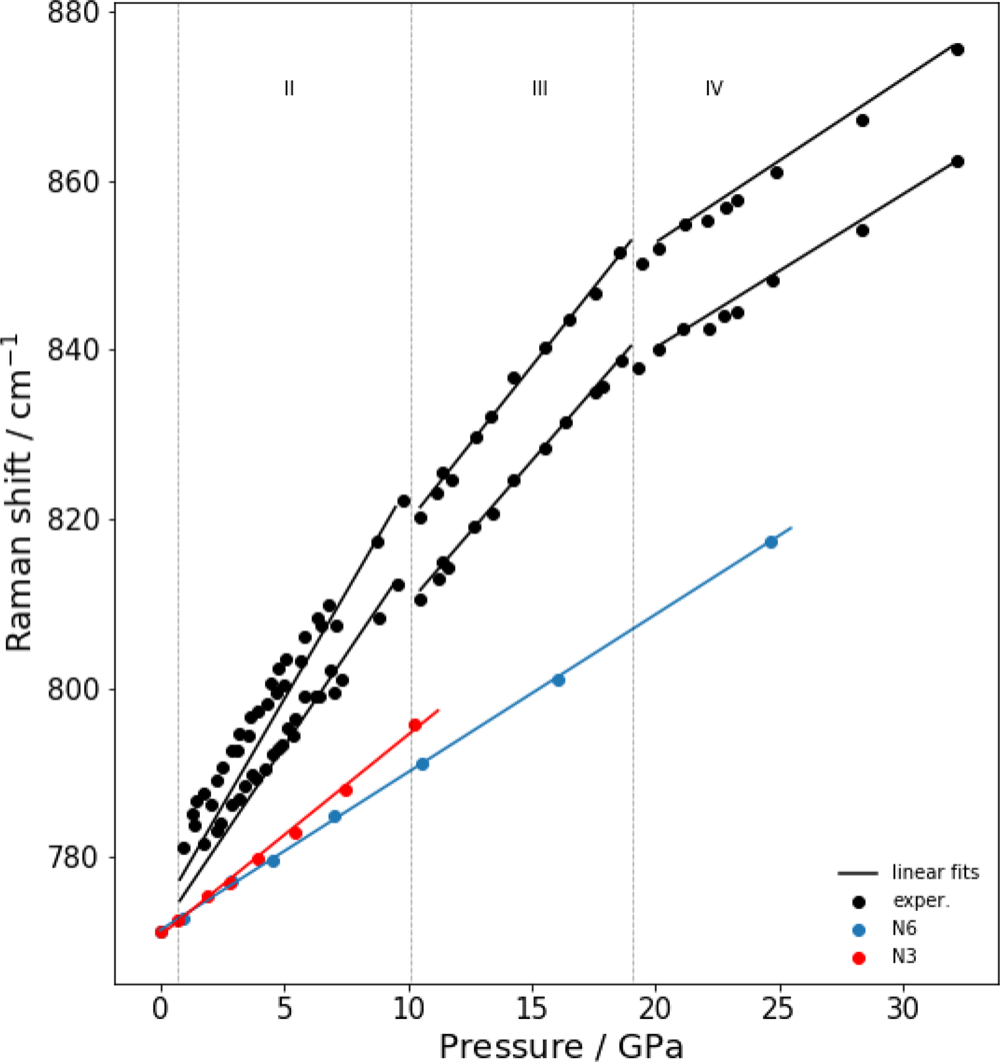
Experimental and computed vibrational frequencies of normal mode ν_1_(*A*_1*g*_). The XP-PCM calculations have been carried out with both *N* = 3 and *N* = 6. The experimental data have been taken by Rademacher et al.^23^

The experimental trend of vibrational frequencies of the *T*_2*g*_, *E*_*g*_, and *A*_1*g*_ modes as a function of pressure is compared with calculations in Figure 6, Figure 7, and Figure 8, respectively. The experimental data refer the measuremet results for phases II, III, and IV, while it has not been reported the vibrational frequencies relative to phase I.

The pressure effect on vibrational frequencies has been analyzed by the numerical calculation of the frequency slope, , which differs for the various normal modes as found experimentally.^23^ The model depends by the choice of *N* parameter as discussed above. However, either *N* parameters provide a semiquantitative description of the experimenatl data, also if the computed values underestimate the experimental results, as can be appreciated in Table 7. A similar behavior has been obtained in studying C_60_ and C_70_ fullerenes under pressure.^16^

**Table 7:** Comparison between Computed (for *N* = 3 and *N* = 6) and Experimental  in a

freq	symm	*N* = 3	*N* = 6	phase II	phase III	phase IV
517.5	*T*_2*g*_	1.98	1.55	2.73 ± 0.47	2.17 ± 0.61	1.18 ± 0.22
662.4	*E*_*g*_	1.92	1.65	3.83 ± 0.31	3.13 ± 0.04	1.58 ± 0.08
771.2	*A*_1*g*_	2.38	1.87	4.64 ± 0.33	3.55 ± 0.22	1.86 ± 0.08

a The experimental data refer to Raman measurements by Rademacher et al.^23^ for the different SF_6_ phases at high pressure.

#### Analysis of Pressure Effects on the Vibrational Frequencies

In this section we present a detailed analysis of the vibrational frequencies as a function of pressure in terms of curvature (direct) and relaxation (indirect) contributions, as given from eq 11 to eq 13. The curvature contribution has been obtained by calculating the vibrational frequencies of SF_6_ keeping the geometry fixed to that of the isolated molecule but reducing the cavity volume to increase the pressure. The  term has been subsequently obtained through a fitting procedure of the vibrational frequencies, which have been reported in Table 8.

**Table 8:** Variations of Vibrational Frequencies (in cm^–1^) with Pressure (GPa) for *N* = 3 and *N* = 6 Parameters^a^

			*N* = 3			
freq	symm	*g̃*_*iij*_				
346.9	*T*_2*u*_	–10.5	2.39	–0.19	0.18	2.58
517.5	*T*_2*g*_	–34.5	1.98	0.38	0.59	1.59
611.3	*T*_1*u*_	–37.0	1.14	0.5	0.64	0.63
662.4	*E*_*g*_	–76.5	1.92	1.22	1.31	0.70
771.2	*A*_1*g*_	–98.5	2.38	1.62	1.70	0.76
978.5	*T*_1*u*_	–108.5	0.65	2.15	1.87	–1.50

			*N* = 6			
freq	symm	*g̃*_*iij*_				
346.9	*T*_2*u*_	–10.5	1.83	–0.12	0.15	1.95
517.5	*T*_2*g*_	–34.5	1.55	0.31	0.47	1.24
611.3	*T*_1*u*_	–37.0	1.00	0.37	0.50	0.63
662.4	*E*_*g*_	–76.5	1.65	0.96	1.04	0.70
771.2	*A*_1*g*_	–98.5	1.87	1.29	1.35	0.58
978.5	*T*_1*u*_	–108.5	1.14	1.61	1.48	–0.47

a Freq and symm refer to normal mode frequencies (in cm^–1^) and symmetries, respectively. *g̃*_*iij*_ represents the cubic force constant (in cm^–1^). *cur* and *rel* subscripts indicate the curvature and relaxation terms, while *anal* and *num* superscripts refer to the calculation approaches.

**Figure 9:**
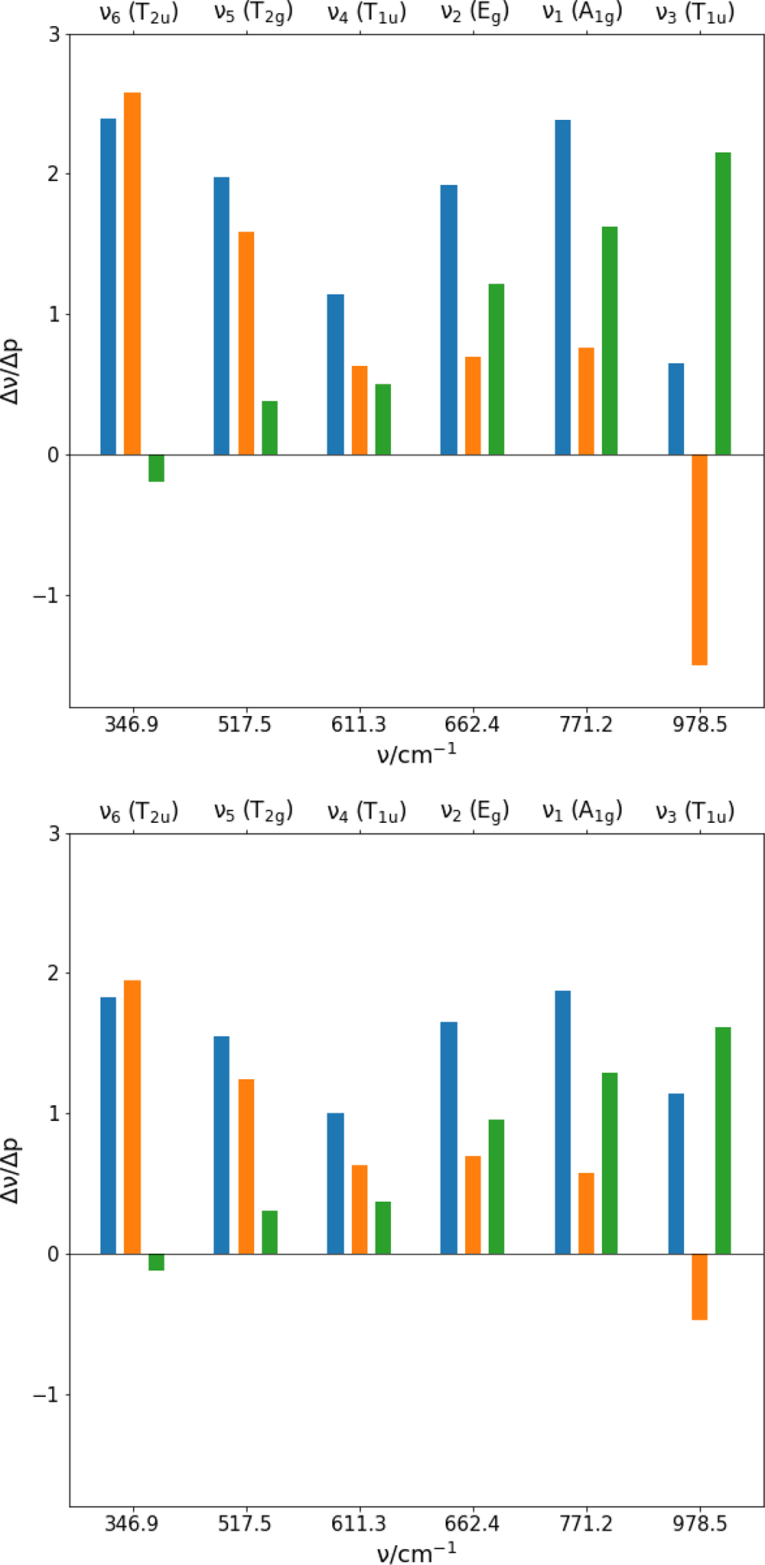
Vibrational analysis in terms of curvature (orange) and relaxation (green) contribution to  (blue). The upper panel refers to the results obtained with *N* = 3, while the lower panel refers to the results obtained with *N* = 6.

The relaxation contribution has been obtained by both the numeric and analytic approaches, described in “Computational Protocol and Details” section. The results with *N* = 3 and *N* = 6 are summarized in Table 8, while a graphic representation is shown in Figure 9.

The results of the vibrational frequency analysis can be summarized considering both the relaxation and curvature contributions. Independent of the value of *N* parameter, the relaxation term, , computed with both numerical and analytic approaches, grows with frequency of the normal modes at the expenses of the curvature term. The results obtained with either approaches, are similar and show that the  is positive with the exception of the lower frequency normal mode with symmetry *T*_2*u*_. The same value is found for ; it can only be noted that for *N* = 6 the value for *T*_2*u*_ mode is negative, but very small. In particular, the relaxation term is related to the anharmonicity through the cubic force constants as discussed in eq 13. In particular, the SF_6_ molecules has only one totally symmetric normal mode (ν_1_ of Figure 2), consequently the relaxation contribution has to be ascribed to the cubic anharmonic constants *g*_*iij*_ in eq 13; the anharmonic constants *g*_*iij*_ are reported in Table 8. The anharmonic character of the normal modes is more pronounced for the higher than for the lower frequency modes and it is more pronounced for stretching modes. This increasing of the anharmonicity is the origin of the growth of the relaxation term with frequencies.

**Figure 10:**
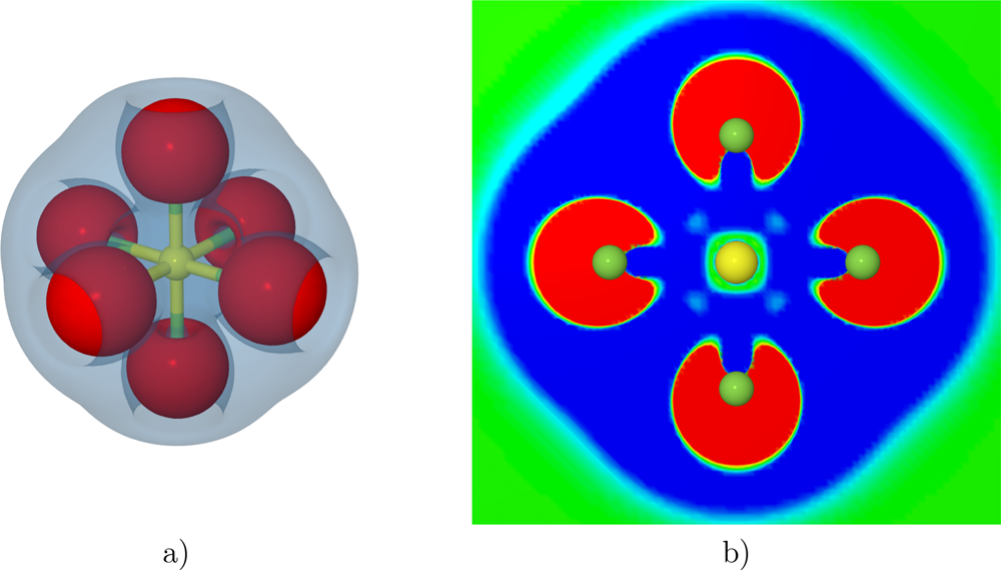
a) Electron density difference between SF_6_ at 0.0 and 24.6 GPa (cutoff ±3 × 10^–5^). b) The red and blue colors on the map represent regions with increase and decrease of electron density, respectively. The isosurface cutoff is ±1.5 × 10^–5^.

The curvature term, , shows positive values with the exception of the highest frequency mode (ν_3_) with symmetry *T*_1*u*_. While the positive values of curvature term present a behavior similar to that found in other systems,^16,17^ the negative value of ν_3_ is rather unusual. The behavior of ν_3_ with pressure has been rationalized considering the Pauli repulsion due to pressure on the electronic structure of SF_6_, especially in the region along the S–F bond. The electron density variation has been analyzed in terms of Mayer bond order,^57,58^ atomic charges,^59−63^ and maps of the electron density difference. The different methods concord in the observed results. In fact, the Mayer bond order decreases with pressure from 0.907 to 0.893 going from 0 to 24.6 GPa with a difference of 0.014; the charges on fluorine atoms increases while that of sulfur decreases (independently by the method, as summarized in Table 9). These results confirm an increase in ionic character of the bond. A similar result has been obtained by the difference of electron density maps depicted in Figure 10. This behavior substantially differs from that found in studying other systems,^13,16,17^ which show an increase of electron density on the bond regions. In the case of SF_6_, the observed electron structure rearrangement with pressure induces a more pronounced variation in the normal modes with asymmetric displacement with respect to the S–F bond.

**Table 9:** S and F Atomic Charges (*e*) Computed through Mulliken,^59−61^ Löwdin,^62^ and Hirshfeld^63^ Partition Schemes^a^

	S			F		
	0.0 GPa	24.6 GPa	Δ	0.0 GPa	24.6 GPa	Δ
Mulliken	1.576	1.617	–0.041	–0.263	–0.270	0.007
Löwdin	1.285	1.303	–0.018	–0.214	–0.217	0.003
Hirshfild	0.598	0.604	–0.006	–0.100	–0.101	0.001

a The differences on atomic charges (Δ) of both S and F atoms have been computed considering the system at the pressure values of 0.0 and 24.6 GPa.

## Conclusions

The XP-PCM method has been applied to compute the structural and vibrational properties of SF_6_ with pressure. The results of the XP-PCM method have been compared with both structural and spectroscopic experimental data, showing a resonable agreement between experiments and calculations. Pressure effects on the vibrational frequencies have been analyzed by considering both the contribution of curvature, , and relaxation, , obtaining useful information for discriminating the importance of confinement and anharmonicity effects. In particular, the curvature term for the ν_3_ normal modes has been rationalized considering the variation of electronic structure of SF_6_ with pressure. In fact, it has been observed that this behavior can be related to the increase of ionic character of the S–F bond under pressure. These results show how the XP-PCM method could provide further useful information for the interpretation of the experimental findings. The different pressure effects in determining a more pronounced covalent or ionic character of bonds in a molecule represent an interesting point to be analyzed in more detail to rationalize the trend of vibrational frequencies with pressure not only using the XP-PCM method but other single molecule computational approaches.^6,64−69^ Finally, it would be useful to extend this kind of analysis in periodic DFT calculations on molecular crystalline systems, which explicitly describe the intermolecular interactions to achieve further information on spectroscopic properties.

## References

[ref1] Schettino V.; Bini R. Constraining Molecules at the Closest Approach: Chemistry at High Pressure. Chem. Soc. Rev. 2007, 36, 869–880. 10.1039/b515964b. 17534474

[ref2] Bini R.; Schettino V. Materials Under Extreme Conditions; Imperial College Press, 2014.

[ref3] Li B.; Ji C.; Yang W.; Wang J.; Yang K.; Xu R.; Liu W.; Cai Z.; Chen J.; Mao H.-k. Diamond anvil cell behavior up to 4 Mbar. Proc. Natl. Acad. Sci. U. S. A. 2018, 115, 1713–1717. 10.1073/pnas.1721425115. 29432145 PMC5828636

[ref4] Cammi R. In Frontiers of Quantum Chemistry; Wójcik M. J., Nakatsuji H., Kirtman B., Ozaki Y., Eds.; Springer Singapore: Singapore, 2018; pp 273–287.

[ref5] Grochala W.; Hoffmann R.; Feng J.; Ashcroft N. The Chemical Imagination at Work in Very Tight Places. Angew. Chem., Int. Ed. 2007, 46, 3620–3642. 10.1002/anie.200602485. 17477335

[ref6] Stauch T. Quantum chemical modeling of molecules under pressure. Int. J. Quantum Chem. 2021, 121, e26208 10.1002/qua.26208. 33350311

[ref7] Chen B.; Hoffmann R.; Cammi R. The Effect of Pressure on Organic Reactions in Fluids–a New Theoretical Perspective. Angew. Chem., Int. Ed. 2017, 56, 11126–11142. 10.1002/anie.201705427. 28738450

[ref8] Cammi R.; Rahm M.; Hoffmann R.; Ashcroft N. W. Varying Electronic Configurations in Compressed Atoms: From the Role of the Spatial Extension of Atomic Orbitals to the Change of Electronic Configuration as an Isobaric Transformation. J. Chem. Theory Comput. 2020, 16, 5047–5056. 10.1021/acs.jctc.0c00443. 32551592 PMC8008388

[ref9] Califano S.; Schettino V.; Neto N. Lattice Dynamics of Molecular Crystals; Springer: Berlin, Heidelberg, 1981.

[ref10] Martin R. M. Electronic Structure: Basic Theory and Practical Methods; Cambridge University Press, 2004.

[ref11] Marx D.; Hutter J. Ab Inito Molecular Dynamics: Basic Theory and Advanced Methods; Cambridge Universty Press, 2009.

[ref12] Wang Y.; Shang S.-L.; fang H.; Liu Z.-K.; Chen L.-Q. First-principles calculations of lattice dynamics and thermal properties of polar solids. npj Comput. Mater. 2016, 2, 16006 10.1038/npjcompumats.2016.6.

[ref13] Cammi R.; Cappelli C.; Mennucci B.; Tomasi J. Calculation and analysis of the harmonic vibrational frequencies in molecules at extreme pressure: Methodology and diborane as a test case. J. Chem. Phys. 2012, 137, 154112 10.1063/1.4757285. 23083153

[ref14] Fukuda R.; Ehara M.; Cammi R. Modeling Molecular Systems at Extreme Pressure by an Extension of the Polarizable Continuum Model (PCM) Based on the Symmetry-Adapted Cluster-Configuration Interaction (SACCI) Method: Confined Electronic Excited States of Furan as a Test Case. J. Chem. Theory Comput. 2015, 11, 2063–2076. 10.1021/ct5011517. 26574410

[ref15] Cammi R. A new extension of the polarizable continuum model: Toward a quantum chemical description of chemical reactions at extreme high pressure. J. Comput. Chem. 2015, 36, 2246–2259. 10.1002/jcc.24206. 26487387

[ref16] Pagliai M.; Cardini G.; Cammi R. Vibrational frequencies of fullerenes C60 and C70 under pressure studied with a quantum chemical model including spatial confinement effects. J. Phys. Chem. A 2014, 118, 5098–5111. 10.1021/jp504173k. 24937194

[ref17] Pagliai M.; Cammi R.; Cardini G.; Schettino V. XP-PCM Calculations of High Pressure Structural and Vibrational Properties of P_4_S_3_. J. Phys. Chem. A 2016, 120, 5136–5144. 10.1021/acs.jpca.6b00590. 26943701

[ref18] Caratelli C.; Cammi R.; Chelli R.; Pagliai M.; Cardini G.; Schettino V. Insights on the Realgar Crystal Under Pressure from XP-PCM and Periodic Model Calculations. J. Phys. Chem. A 2017, 121, 8825–8834. 10.1021/acs.jpca.7b08868. 29083904

[ref19] Tomasi J.; Persico M. Molecular Interactions in Solution: An Overview of Methods Based on Continuous Distributions of the Solvent. Chem. Rev. 1994, 94, 2027–2094. 10.1021/cr00031a013.

[ref20] Tomasi J.; Mennucci B.; Cammi R. Quantum Mechanical Continuum Solvation Models. Chem. Rev. 2005, 105, 2999–3094. 10.1021/cr9904009. 16092826

[ref21] Labet V.; Hoffmann R.; Ashcroft N. W. A Fresh Look at Dense Hydrogen under Pressure. II. Chemical and Physical Models Aiding our Understanding of Evolving H-H Separations. J. Chem. Phys. 2012, 136, 074502 10.1063/1.3679736. 22360243

[ref22] Salvi P.; Schettino V. Infrared and Raman spectra and phase transition of the SF_6_ crystal. Anharmonic interactions and two-phonon infrared absorption. Chem. Phys. 1979, 40, 413–424. 10.1016/0301-0104(79)85154-X.

[ref23] Rademacher N.; Friedrich A.; Morgenroth W.; Bayarjargal L.; Milman V.; Winkler B. High-pressure phases of SF6 up to 32 GPa from X-ray diffraction and Raman spectroscopy. J. Phys. Chem. Solids 2015, 80, 11–21. 10.1016/j.jpcs.2014.12.016.

[ref24] Dows D. A.; Wieder G. M. Infrared intensities in crystalline SF_6_. Spectrochim. Acta 1962, 18, 1567–1574. 10.1016/S0371-1951(62)80284-7.

[ref25] Rubin B.; McCubbin T.K.; Polo S.R. Vibrational Raman Spectrum of SF_6_. J. Mol. Spectrosc. 1978, 69, 254–259. 10.1016/0022-2852(78)90063-2.

[ref26] Sasaki S.; Tomida Y.; Shimizu H. High-Pressure Raman Study of Sulfur Hexafluoride up to 10 GPa. J. Phys. Soc. Jpn. 1992, 61, 514–518. 10.1143/JPSJ.61.514.

[ref27] Murnaghan F. D. The Compressibility of Media under Extreme Pressures. Proc. Natl. Acad. Sci. U. S. A. 1944, 30, 244–247. 10.1073/pnas.30.9.244. 16588651 PMC1078704

[ref28] Alvarez S. A cartography of the van der Waals territories. Dalton Trans. 2013, 42, 8617–8636. 10.1039/c3dt50599e. 23632803

[ref29] Rahm M.; Ångqvist M.; Rahm J. M.; Erhart P.; Cammi R. Non-Bonded Radii of the Atoms Under Compression. ChemPhysChem 2020, 21, 2441–2453. 10.1002/cphc.202000624. 32896974

[ref30] Moroni L.; Ceppatelli M.; Gellini C.; Salvi P. R.; Bini R. Excitation of Crystalline All Trans Retinal under Pressure. Phys. Chem. Chem. Phys. 2002, 4, 5761–5767. 10.1039/B207312A.

[ref31] Cockcroft J. K.; Fitch A. N. The solid phases of sulphur hexafluoride by powder neutron diffraction. Z. Kristallogr. 1988, 184, 123–145. 10.1524/zkri.1988.184.1-2.123.

[ref32] Dolling G.; Powell B.; Sears V. Neutron diffraction study of the plastic phases of polycrystalline SF6 and CBr4. Mol. Phys. 1979, 37, 1859–1883. 10.1080/00268977900101381.

[ref33] Dove M. T.; Pawley G. S. A molecular dynamics simulation study of the plastic crystalline phase of sulphur hexafluoride. J. Phys. C: Solid State Phys. 1983, 16, 5969–5983. 10.1088/0022-3719/16/31/012.

[ref34] Dove M. T.; Pawley G. S. A molecular dynamics simulation study of the orientationally disordered phase of sulphur hexafluoride. J. Phys. C: Solid State Phys. 1984, 17, 6581–6599. 10.1088/0022-3719/17/36/014.

[ref35] Dove M.; Tucker M.; Keen D. Neutron total scattering method: simultaneous determination of long-range and short-range order in disordered materials. Eur. J. Mineral. 2002, 14, 331–348. 10.1127/0935-1221/2002/0014-0331.

[ref36] Spitznagel G. W.; Clark T.; Schleyer P. v.; Hehre W. J. An Evaluation of the Performance of Diffuse Function-Augmented Basis Sets for Second Row Elements, Na-Cl. J. Comput. Chem. 1987, 8, 1109–1116. 10.1002/jcc.540080807.

[ref37] Frisch M. J.; Trucks G. W.; Schlegel H. B.; Scuseria G. E.; Robb M. A.; Cheeseman J. R.; Scalmani G.; Barone V.; Mennucci B.; Petersson G. A.; Gaussian’09 Revision C.01; Gaussian Inc.: Wallingford, CT, 2010.

[ref38] Bartell L.; Doun S. Structures of hexacoordinate compounds of main-group elements: Part III. An electron diffraction study of SF6. J. Mol. Struct. 1978, 43, 245–249. 10.1016/0022-2860(78)80010-6.

[ref39] Hohenberg P.; Kohn W. Inhomogeneous Electron Gas. Phys. Rev. 1964, 136, B864–B871. 10.1103/PhysRev.136.B864.

[ref40] Kohn W.; Sham L. J. Self-Consistent Equations Including Exchange and Correlation Effects. Phys. Rev. 1965, 140, A1133–A1138. 10.1103/PhysRev.140.A1133.

[ref41] Slater J. C. The Self-Consistent Field for Molecular and Solids, Quantum Theory of Molecular and Solids; McGraw-Hill: New York, 1974; Vol. 4.

[ref42] Vosko S. H.; Wilk L.; Nusair M. Accurate spin-dependent electron liquid correlation energies for local spin density calculations: a critical analysis. Can. J. Phys. 1980, 58, 1200–1211. 10.1139/p80-159.

[ref43] Becke A. D. Density-functional exchange-energy approximation with correct asymptotic behavior. Phys. Rev. A: At., Mol., Opt. Phys. 1988, 38, 3098–3100. 10.1103/PhysRevA.38.3098. 9900728

[ref44] Lee C.; Yang W.; Parr R. Development of the Colle-Salvetti Correlation-Energy Formula into a Functional of the Electron-Density. Phys. Rev. B: Condens. Matter Mater. Phys. 1988, 37, 785–789. 10.1103/PhysRevB.37.785. 9944570

[ref45] Becke A. D. Density-Functional Thermochemistry. III. The Role of Exact Exchange. J. Chem. Phys. 1993, 98, 5648–5652. 10.1063/1.464913.

[ref46] Yanai T.; Tew D. P.; Handy N. C. A new hybrid exchange-correlation functional using the Coulomb-attenuating method (CAM-B3LYP). Chem. Phys. Lett. 2004, 393, 51–57. 10.1016/j.cplett.2004.06.011.

[ref47] Cohen A. J.; Handy N. C. Dynamic correlation. Mol. Phys. 2001, 99, 607–615. 10.1080/00268970010023435.

[ref48] Perdew J. P.; Burke K.; Ernzerhof M. Generalized Gradient Approximation Made Simple. Phys. Rev. Lett. 1996, 77, 3865–3868. 10.1103/PhysRevLett.77.3865. 10062328

[ref49] Perdew J. P.; Burke K.; Ernzerhof M. Generalized Gradient Approximation Made Simple [Phys. Rev. Lett. 77, 3865 (1996)]. Phys. Rev. Lett. 1997, 78, 1396–1396. 10.1103/PhysRevLett.78.1396. 10062328

[ref50] Adamo C.; Barone V. Toward Reliable Density Functional Methods without Adjustable Parameters: The PBE0Model. J. Chem. Phys. 1999, 110, 6158–6169. 10.1063/1.478522.

[ref51] Chai J.-D.; Head-Gordon M. Long-range corrected hybrid density functionals with damped atom-atom dispersion corrections. Phys. Chem. Chem. Phys. 2008, 10, 6615–6620. 10.1039/b810189b. 18989472

[ref52] Cammi R.; Verdolino V.; Mennucci B.; Tomasi J. Towards the Elaboration of a QM Method to Describe Molecular Solutes under Effect of a Very High Pressure. Chem. Phys. 2008, 344, 135–141. 10.1016/j.chemphys.2007.12.010.

[ref53] Herzberg G. Molecular Spectra and Molecular Structure II. Infrared and Raman Spectra of Polyatomic Molecules; Krieger: Malabar, FL, 1991.

[ref54] Edelson D.; McAfee K. Note on the infrared spectrum of sulfur hexafluoride. J. Chem. Phys. 1951, 19, 1311–1312. 10.1063/1.1748022.

[ref55] Wagner N. L.; Wüest A.; Christov I. P.; Popmintchev T.; Zhou X.; Murnane M. M.; Kapteyn H. C. Monitoring molecular dynamics using coherent electrons from high harmonic generation. Proc. Natl. Acad. Sci. U. S. A. 2006, 103, 13279–13285. 10.1073/pnas.0605178103. 16895984 PMC1533881

[ref56] Birch F. Finite Elastic Strain of Cubic Crystals. Phys. Rev. 1947, 71, 809–824. 10.1103/PhysRev.71.809.

[ref57] Mayer I. Charge bond order and valence in the AB initio SCF theory. Chem. Phys. Lett. 1983, 97, 270–274. 10.1016/0009-2614(83)80005-0.

[ref58] Mayer I. On bond orders and valences in the Ab initio quantum chemical theory. Int. J. Quantum Chem. 1986, 29, 73–84. 10.1002/qua.560290108.

[ref59] Mulliken R. S. Electronic Population Analysis on LCAO-MO Molecular Wave Functions. I. J. Chem. Phys. 1955, 23, 1833–1840. 10.1063/1.1740588.

[ref60] Mulliken R. S. Electronic Population Analysis on LCAOMO Molecular Wave Functions. II. Overlap Populations, Bond Orders, and Covalent Bond Energies. J. Chem. Phys. 1955, 23, 1841–1846. 10.1063/1.1740589.

[ref61] Mulliken R. S. Criteria for the Construction of Good Self-Consistent-Field Molecular Orbital Wave Functions, and the Significance of LCAO-MO Population Analysis. J. Chem. Phys. 1962, 36, 3428–3439. 10.1063/1.1732476.

[ref62] Löwdin Per-Olov On the Non-Orthogonality Problem Connected with the Use of Atomic Wave Functions in the Theory of Molecules and Crystals. J. Chem. Phys. 1950, 18, 365–375. 10.1063/1.1747632.

[ref63] Hirshfeld F. Bonded-atom fragments for describing molecular charge densities. Theoret. Chim. Acta 1977, 44, 129–138. 10.1007/BF00549096.

[ref64] Subramanian G.; Mathew N.; Leiding J. A generalized force-modified potential energy surface for mechanochemical simulations. J. Chem. Phys. 2015, 143, 134109 10.1063/1.4932103. 26450294

[ref65] Zaleśny R.; Góra R. W.; Luis J. M.; Bartkowiak W. On the particular importance of vibrational contributions to the static electrical properties of model linear molecules under spatial confinement. Phys. Chem. Chem. Phys. 2015, 17, 21782–21786. 10.1039/C5CP02865E. 26247540

[ref66] Spooner J.; Smith B.; Weinberg N. Effect of high pressure on the topography of potential energy surfaces. Can. J. Chem. 2016, 94, 1057–1064. 10.1139/cjc-2016-0295.

[ref67] Stauch T. A mechanochemical model for the simulation of molecules and molecular crystals under hydrostatic pressure. J. Chem. Phys. 2020, 153, 134503 10.1063/5.0024671. 33032415

[ref68] Chouj M.; Basiak B.; Bartkowiak W. Partitioning of the interaction-induced polarizability of molecules in helium environments. Int. J. Quantum Chem. 2021, 121, e26544 10.1002/qua.26544.

[ref69] Scheurer M.; Dreuw A.; Epifanovsky E.; Head-Gordon M.; Stauch T. Modeling Molecules under Pressure with Gaussian Potentials. J. Chem. Theory Comput. 2021, 17, 583–597. 10.1021/acs.jctc.0c01212. 33350311

